# Supplementary material for: Elastic dosage compensation by X-chromosome upregulation
Source: Nat Commun. 2022 Apr 6;13:1854. doi: 10.1038/s41467-022-29414-1 (PMC8987076; doi:10.1038/s41467-022-29414-1)
Supplement: Supplementary file 1 — Supplementary Information [file 41467_2022_29414_MOESM1_ESM.pdf]

SUPPLEMENTARY INFORMATION

Elastic dosage compensation by X-chromosome upregulation

Antonio Lentini[1], Huaitao Cheng[2], Joyce C. Noble[1], Natali Papanicolaou[1], Christos Coucoravas[1], Nathanael Andrews[2], Qiaolin Deng[3], Martin Enge[2] and Björn Reinius[1]*

[1]Department of Medical Biochemistry and Biophysics, Karolinska Institutet, Stockholm, Sweden.

[2]Department of Oncology and Pathology, Karolinska Institutet, Stockholm, Sweden.

[3]Department of Physiology and Pharmacology, Karolinska Institutet, Stockholm, Sweden.

*Correspondence to: bjorn.reinius@ki.se

Supplementary Figure 1 –5

Other Supplementary Materials for this manuscript includes the following:

Supplementary Data 1 (Microsoft Excel format).

Supplementary Data 2 (Microsoft Excel format).

Supplementary Fig. 1

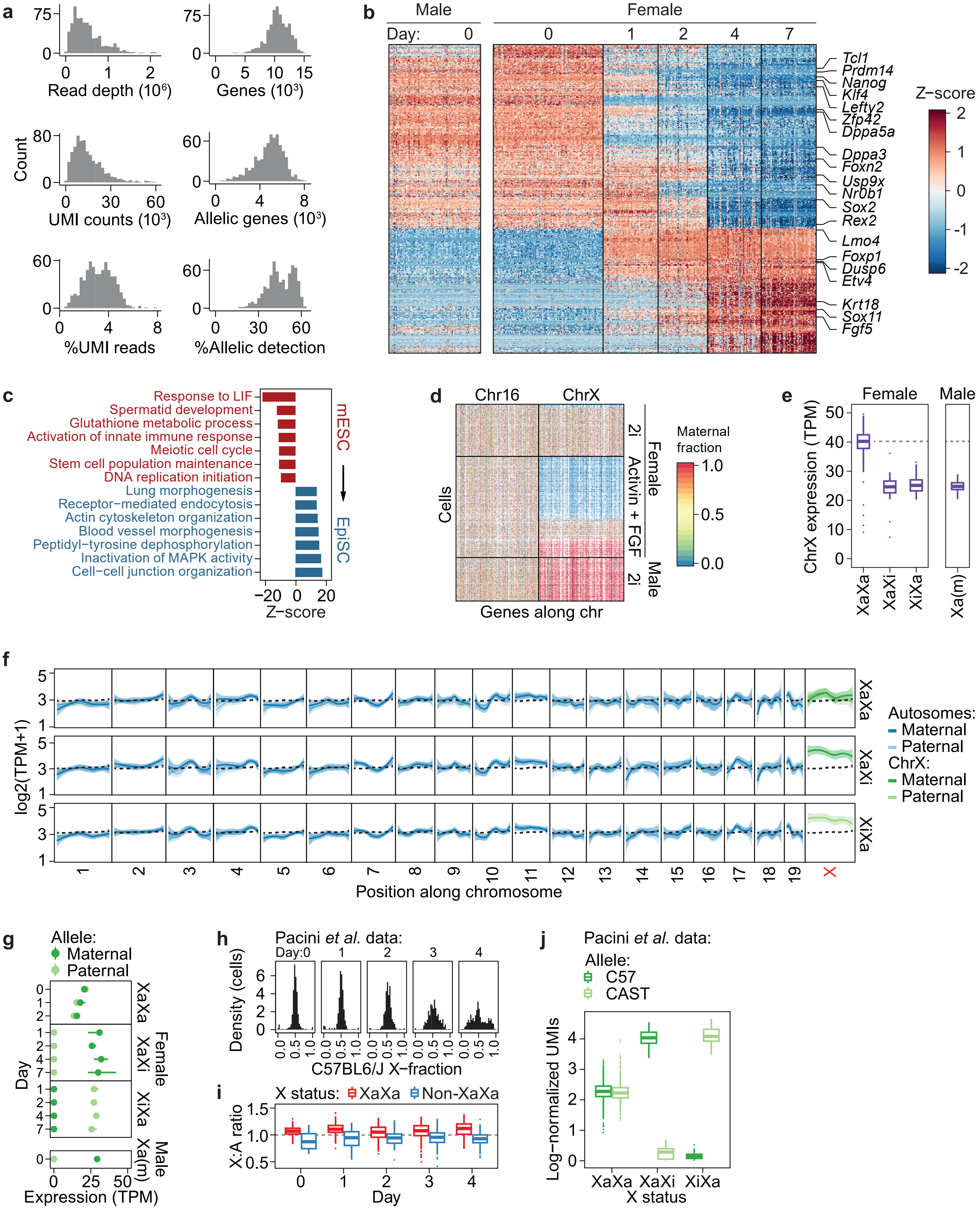

Supplementary Fig. 1. Validation of X-upregulation upon exit from pluripotency.
a. Histograms of read and gene metrics for generated Smart-seq3 data.
b. Heatmap of differentially expressed genes along mESC (day 0) priming towards EpiSCs (day 1-7). See also **Supplementary Data 2**.
c. GO term enrichment for genes in **b.** See also **Supplementary Data 2**.
d. Heatmap of maternal fractions for chr16 and chrX for genes detected in >10% of cells for mESCs under 2i or EpiSC priming conditions.
e. Boxplot showing total expression (RNA output of both alleles) for X-linked genes (n = 1,158–1,337) grouped by XCI state (n = 675 cells total). Dashed line indicates the XaXa median. Data shown as median, first and third quartiles, and 1.5x inter-quartile range (IQR).
f. Rolling average (LOESS fit ± 95% confidence interval) of allelic gene expression along intra-chromosomal coordinates (x-axis) for female mESCs cells cultured in EpiSC priming conditions grouped by XCI state. Dashed line indicates the average for autosomes.
g. Allele-resolved chrX expression grouped by sex, XCI state and timepoints of EpiSC priming, shown as median ± 95% confidence interval.
h. Density plots of allelic ratios for mESCs cultured under serum/LIF conditions for up to 4 days.
i. Boxplots of X:Autosomal ratios for cells in **j**, grouped by XCI state. Data shown as median, first and third quartiles, and 1.5x IQR.
j. Boxplots of allelic expression for cells in **j**, grouped by XCI state. Data shown as median, first and third quartiles, and 1.5x IQR.

Supplementary Fig. 2

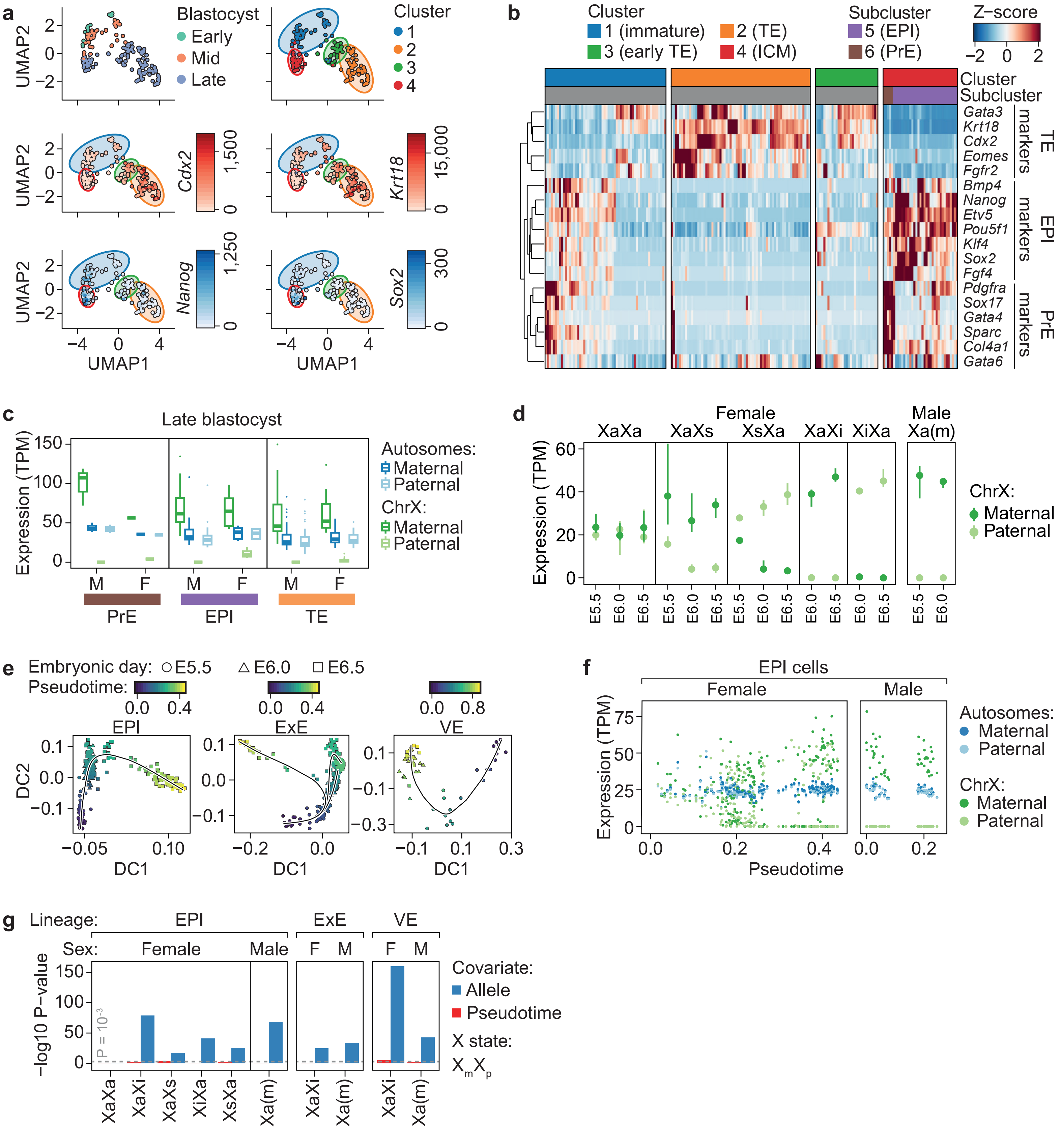

Supplementary Fig. 2. Extended analyses of early embryo development.
a. UMAP dimensionality reduction for pre-implantation blastocysts using the top 1,000 variable genes and graph-based clustering of blastocysts. Trophectoderm- (TE) and inner cell mass (ICM) marker genes shown in red and blue, respectively.
b. Heatmap and hierarchical clustering of pre-implantation blastocysts using lineage-specific marker genes for trophectoderm (TE), epiblast (EPI), primitive endoderm (PrE) lineages.
c. Boxplots of allelic expression of late blastocyst subclusters identified in (**a-b**). Data shown as median, first and third quartiles, and 1.5x IQR.
d. Allelic ChrX expression of post-implantation epiblast (EPI) cells grouped by embryonic day and XCI state, shown as median ± 95% confidence interval.
e. Diffusion map dimensionality reduction and Slingshot trajectory inference per post-implantation lineage. Embryonic day indicated as E5.5 (circle), E6.0 (triangle) or E6.5 (square).
f. Allelic expression along pseudotime trajectories from **e** for post-implantation EPI cells.
g. Association of allele usage or pseudotime on allelic expression using linear modelling.

Supplementary Fig. 3

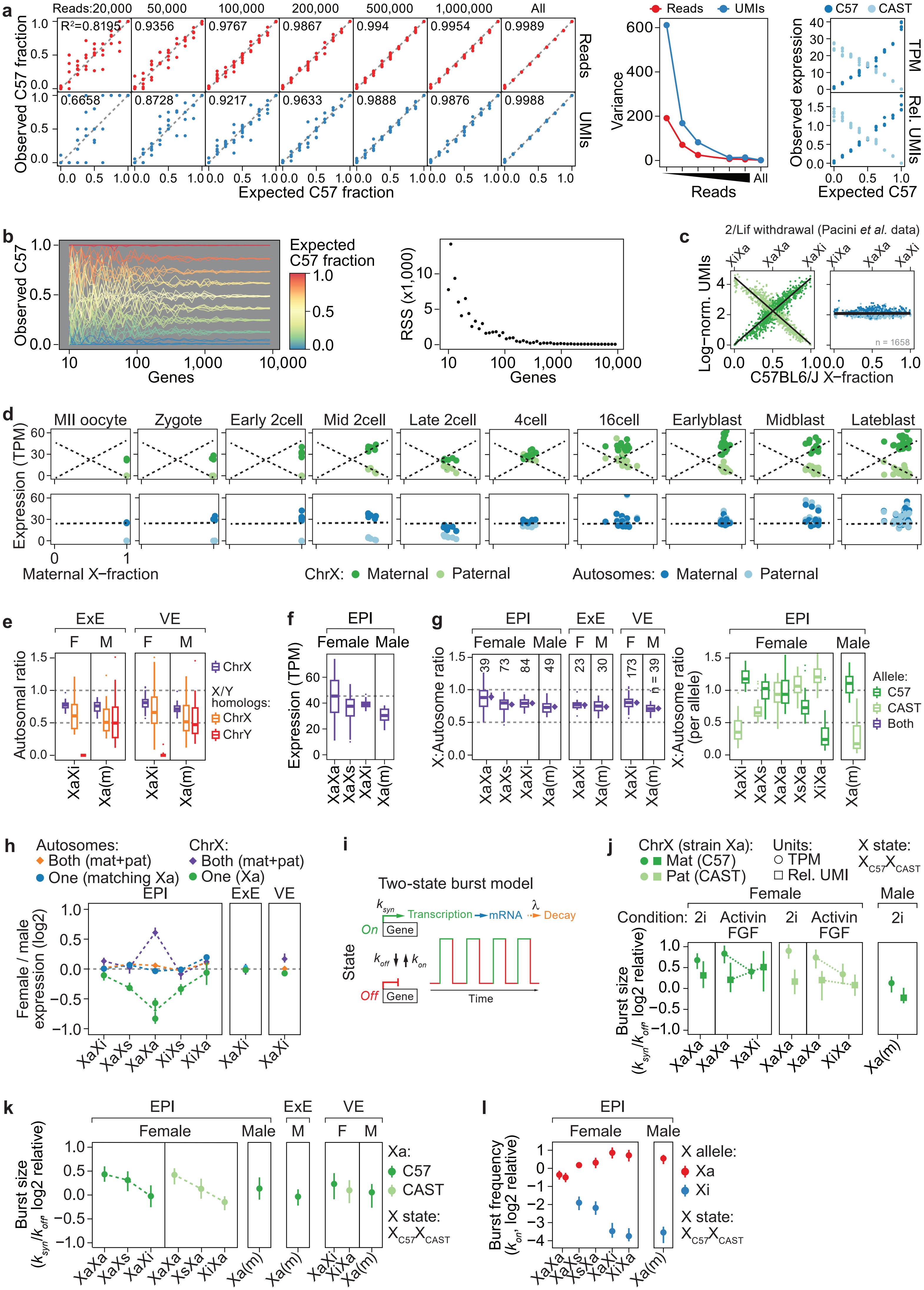

Supplementary Fig. 3. Extended analyses of relationship between X-upregulation and X-inactivation.
a. Accuracy of allelic inference of Smart-seq3 using experimentally controlled ratios of C57:CAST RNA, based on read count down sampling. R^2 and variance (σ^2) calculated from linear model.
b. Stability of allelic inference of Smart-seq3 based on gene subsampling. RSS = residual sum of squares.
c. Scatter plot of expression level per allele (y-axis) and maternal X-fraction per female cell (x-axis) for mESCs cultured under serum/LIF conditions for up to 4 days (n = 1,658 cells; Pacini et al. 2021) with lines indicating linear model mean ± 95% confidence interval. Shown for autosomes (blue; n = 1,074–4,913 genes) and chrX (green; n = 23–182). Legend as in panel **d.** but for C57 and CAST alleles instead of maternal and paternal, respectively.
d. Relationship between X expression and maternal X-fraction in pre-implantation embryos, dashed lines represent post-implantation EPI trends.
e. Autosomal ratios per cell shown as box plots grouped by lineage, sex, and XCI state for chrX (n = 129–410 genes) and ancestral X-Y homologs (n = 5–9 genes) separately. X alleles indicated as active (Xa) or inactive (Xi). Data shown as median, first and third quartiles, and 1.5x IQR.
f. Boxplots of total chrX expression (accumulative RNA output of both alleles) in EPI cells grouped by XCI state. Data shown as median, first and third quartiles, and 1.5x IQR.
g. X:Autosomal ratios shown as box plots or bootstrapped median (diamond ± 95% confidence) interval for total expression (both alleles; left) or allelic resolution (right). Data stratified by lineage, sex and XCI state. F = female, M = male. Data shown as median, first and third quartiles, and 1.5x IQR.
h Female:male expression ratios calculated either by total expression (accumulative from both alleles; diamond) or for one active X allele (Xa; dot), shown as median ± 95% confidence interval. Data representative of n = 392 cells total. For epiblasts (EPI) the data points are stratified according to female rXCI state (x-axis). Autosomal genes (n = 14,941–17,643). X-linked genes (n = 597–713) genes.
i. Inference of transcriptional kinetics from a two-state model of transcription (see Larsson et al. 2019 for details).
j. Transcriptional burst size (k_{syn}/k_{off}) for active X alleles (Xa) grouped by sex, culture condition and XCI state, shown as median ± 95% confidence interval, inferred by either TPM (dot) or relative UMIs (square). The data is shown relative to median autosomal burst size.
k. Transcriptional burst size (k_{syn}/k_{off}) for active X alleles (Xa) grouped by sex, lineage and rXCI status, shown as median ± 95% confidence interval. The data is shown relative to median autosomal burst size. Note however that the accuracy of burst-size inference is limited in non-UMI (Smart-seq2) scRNA-seq data (See Larsson et al. 2019 for details).
l. Transcriptional burst frequency (k_{on}) for Xa and Xi alleles in EPI cells grouped by sex and rXCI status, shown as median ± 95% confidence interval. The data is shown relative to median autosomal burst frequency.

Supplementary Fig. 4

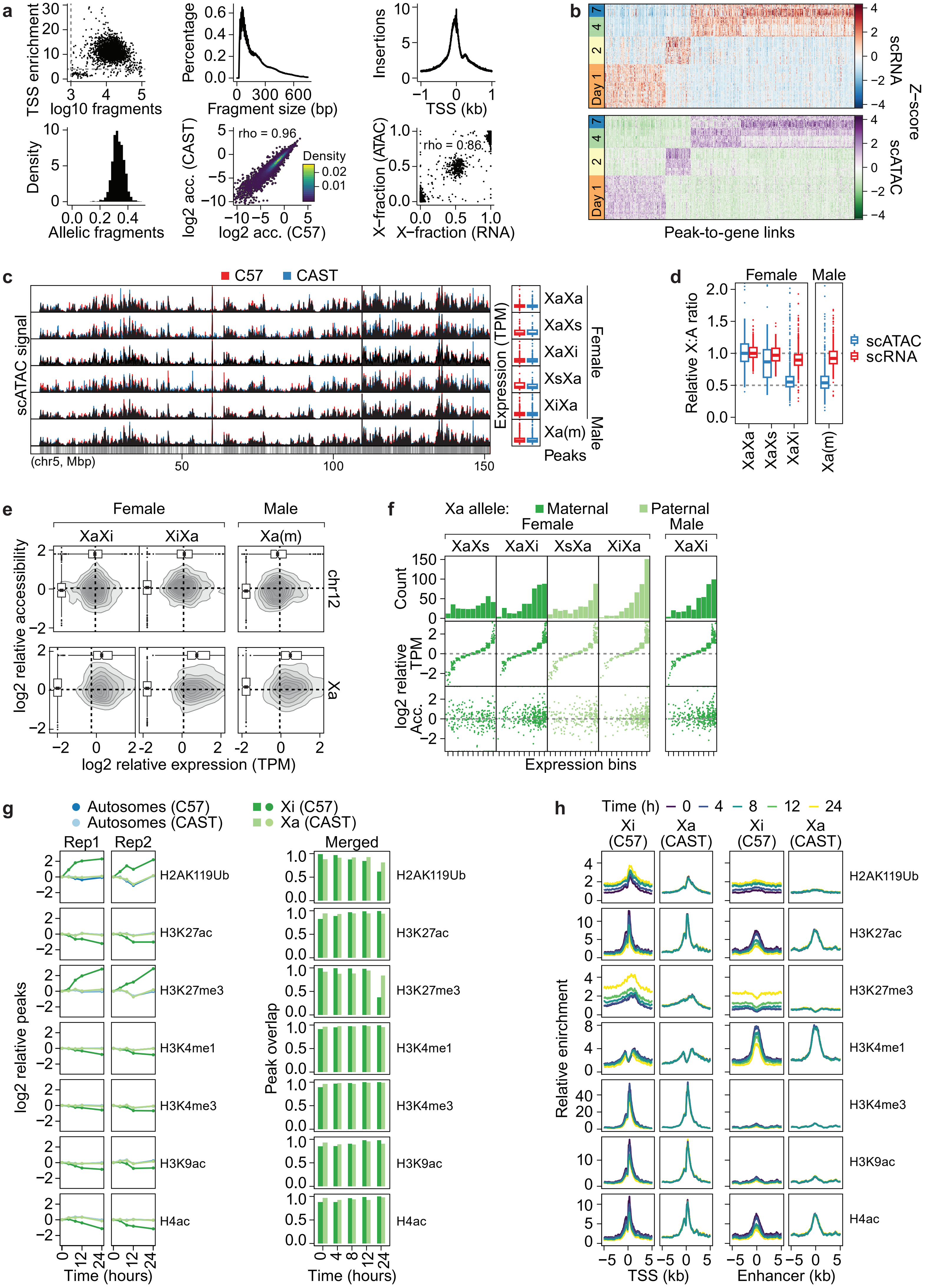

Supplementary Fig. 4. Extended analyses of genomic regulation of X-upregulation.
a. Quality metrics for generated combined scRNA/ATAC-seq data.
b. Heatmaps of linked accessibility and expression along EpiSC priming.
c. Genome tracks of allelic accessibility for an autosome (chr5), grouped by X-inactivation (XCI) state. Corresponding allele-resolved expression is shown to the right as median, first and third quartiles, and 1.5x IQR.
d. Boxplots of X:Autosomal ratios relative to XaXa cells for expression (scRNA) and accessibility (scATAC). Data shown as median, first and third quartiles, and 1.5x IQR.
e. 2D density plots of gene-level accessibility (y-axis) and expression (x-axis) relative to XaXa cells shown per sex and XCI state for the active X allele (Xa) and chr12 (allele corresponding to Xa). Dashed line indicates autosomal median per group.
f. Same data as in **e** but shown as jitter plots binned by relative expression.
g. Number of native ChIP-seq peaks per modification and allele relative to the 0h timepoint (left) and peak overlap with all other timepoints per timepoint (right).
h. Normalized allelic enrichment profiles of histone modifications for Xa and Xi alleles shown relative to transcription start site (TSS; left) or enhancers (right).

Supplementary Fig. 5

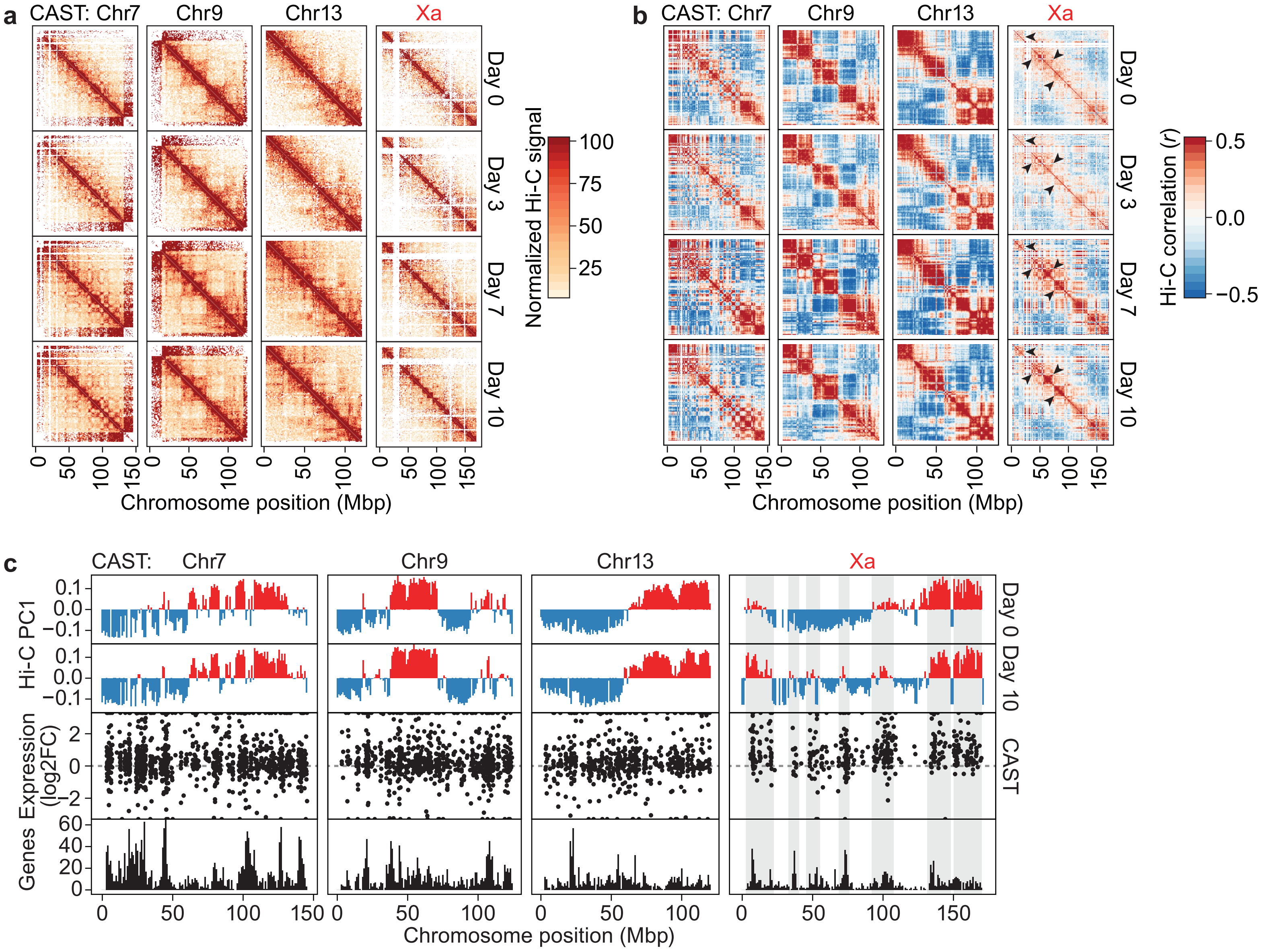

Supplementary Fig. 5. Extended analyses of chromatin contacts.
a. Normalized *in situ* Hi-C contact signal maps for Xa and representative autosomes along mESC differentiation at 1Mb resolution.
b. Same as **a** but shown as contact correlation maps.
c. Eigenvector plots of chromosome compartmentalization. Also shown is Smart-seq3 relative expression of the CAST allele (XiXa *vs.* XaXa).
