# Supplementary material for: Elastic dosage compensation by X-chromosome upregulation
Source: Nat Commun. 2022 Apr 6;13:1854. doi: 10.1038/s41467-022-29414-1 (PMC8987076; doi:10.1038/s41467-022-29414-1)
Supplement: Supplementary file 3 — Description of Additional Supplementary Files [file 41467_2022_29414_MOESM3_ESM.pdf]

Description of Additional Supplementary Files

File Name: Supplementary Data 1
Description: Sample annotations for all cells captured in Smart-seq3 and Smart3-ATAC experiments.

File Name: Supplementary Data 2
Description: Differentially expressed genes along mESC differentiation towards EpiSCs and gene ontology (GO) term enrichment.
