# Supplementary material for: Elastic dosage compensation by X-chromosome upregulation
Source: Nat Commun. 2022 Apr 6;13:1854. doi: 10.1038/s41467-022-29414-1 (PMC8987076; doi:10.1038/s41467-022-29414-1)
Supplement: Supplementary file 6 — Reporting Summary [file 41467_2022_29414_MOESM6_ESM.pdf]

nature portfolio

Corresponding author(s): Björn Reinius

Last updated by author(s): Jan 20, 2022

Reporting Summary

Nature Portfolio wishes to improve the reproducibility of the work that we publish. This form provides structure for consistency and transparency in reporting. For further information on Nature Portfolio policies, see our Editorial Policies and the Editorial Policy Checklist.

Statistics

For all statistical analyses, confirm that the following items are present in the figure legend, table legend, main text, or Methods section.

n/a	Confirmed	
☐	☒	The exact sample size (*n*) for each experimental group/condition, given as a discrete number and unit of measurement
☐	☒	A statement on whether measurements were taken from distinct samples or whether the same sample was measured repeatedly
☐	☒	The statistical test(s) used AND whether they are one- or two-sided<br>*Only common tests should be described solely by name; describe more complex techniques in the Methods section.*
☐	☒	A description of all covariates tested
☐	☒	A description of any assumptions or corrections, such as tests of normality and adjustment for multiple comparisons
☐	☒	A full description of the statistical parameters including central tendency (e.g. means) or other basic estimates (e.g. regression coefficient) AND variation (e.g. standard deviation) or associated estimates of uncertainty (e.g. confidence intervals)
☒	☐	For null hypothesis testing, the test statistic (e.g. *F*, *t*, *r*) with confidence intervals, effect sizes, degrees of freedom and *P* value noted<br>*Give P values as exact values whenever suitable.*
☒	☐	For Bayesian analysis, information on the choice of priors and Markov chain Monte Carlo settings
☒	☐	For hierarchical and complex designs, identification of the appropriate level for tests and full reporting of outcomes
☐	☒	Estimates of effect sizes (e.g. Cohen's *d*, Pearson's *r*), indicating how they were calculated

Our web collection on statistics for biologists contains articles on many of the points above.

Software and code

Policy information about availability of computer code

Data collection	bcl2fastq (v2.20.0.422) was used to convert base calls into FASTQ files.
Data analysis	Data processing was performed with zUMIs (v2.8.0), STAR (v2.7.2a), SNPsplit (v0.3.2), fastp (v0.20.0), bowtie2 (v2.4.1), biobambam2 (v2.0.87), Salmon (v0.14.1), MACS2 (2.1.2), deepTools (v3.3.0), HiCUP (v0.8.0), juicer tools (v1.22.01) and R (v3.6.1) using custom R scripts involving the R packages ArchR (v1.0.1), Biobase (v2.44.0), BiocGenerics (v0.30.0), BiocParallel (v1.18.1), biomaRt (v2.40.3), boot (v1.3-23), circlize (v0.4.6), ComplexHeatmap (v2.0.0), cowplot (v1.0.0), data.table (v1.12.2), DelayedArray (v0.10.0), DESeq2 (v1.24.0), GenomeInfoDb (v1.20.0), GenomicRanges (v1.36.0), ggbeeswarm (v0.6.0), ggplot2 (v3.2.1), hdf5r (v1.3.2.9000), IRanges (v2.18.1), loomR (v0.2.1.9000), magrittr (v1.5), MAST (v1.10.0), matrixStats (v0.54.0), mclust (v5.4.5), princurve (v2.1.4), R6 (v2.4.0), RColorBrewer (v1.1-2), rtracklayer (v1.44.2), S4Vectors (v0.22.0), scater (v1.12.2), scran (v1.12.1), SingleCellExperiment (v1.6.0), slingshot (v1.2.0), SummarizedExperiment (v1.14.1), tximport (v1.12.3). Data and code generated during this study are available at https://github.com/reiniuslab/Lentini_XCU_in_vivo.

For manuscripts utilizing custom algorithms or software that are central to the research but not yet described in published literature, software must be made available to editors and reviewers. We strongly encourage code deposition in a community repository (e.g. GitHub). See the Nature Portfolio guidelines for submitting code & software for further information.

Data

Policy information about availability of data

All manuscripts must include a data availability statement. This statement should provide the following information, where applicable:

- Accession codes, unique identifiers, or web links for publicly available datasets
- A description of any restrictions on data availability
- For clinical datasets or third party data, please ensure that the statement adheres to our policy

Raw and pre-processed data generated is publicly available at ArrayExpress under accession E-MTAB-9324 (Smart-seq3), E-MTAB-10709 (Allelic dilution series) and E-MTAB-10714 (Combined Smart-seq3+scATAC). Previously published raw data is available at Gene Expression Omnibus under accessions GSE45719, GSE74155, GSE109071, GSE116480, GSE23943, GSE80810, GSE90516, GSE116649 and GSE151009. C57BL6/J reference genome (GRCm38_68.fa) and strain-specific SNPs (mgp.v5.merged.snps_all.dbSNP142.vcf.gz) were obtained from the Mouse Genomes Project (https://www.sanger.ac.uk/data/mouse-genomes-project/), and gene annotations (Mus_musculus.GRCm38.97.chr.gtf) were obtained from ENSEMBL (https://www.ensembl.org/Mus_musculus/Info/Index). Protein-coding transcript sequences (gencode.vM22.pc_transcripts.fa) was obtained from GENCODE (https://www.gencodegenes.org/mouse/release_M22.html).

Field-specific reporting

Please select the one below that is the best fit for your research. If you are not sure, read the appropriate sections before making your selection.

☒ Life sciences ☐ Behavioural & social sciences ☐ Ecological, evolutionary & environmental sciences

For a reference copy of the document with all sections, see nature.com/documents/nr-reporting-summary-flat.pdf

Life sciences study design

All studies must disclose on these points even when the disclosure is negative.

Sample size	No sample size calculation performed. Sample sizes were selected to yield sufficient cell numbers per condition (n > 20) to allow kinetic inference.
Data exclusions	Single-cell RNA-seg libraries of low read depth (>3 MADs) were removed for any further analysis.
Replication	The XCU dynamic were replicated in two in vitro conditions of embryonic stem cell priming (Activin/FGF, and 2i/Lif withdrawal) and further observed during embryonic development in vivo (mouse pre- and peri-implantation development). All attempts at replicating our findings were successful using the data generated herein or using publicly available datasets. Replication of findings from public allele-specific ChIP-seq and Hi-C data was not possible due to lack of comparable datasets of high quality.
Randomization	Cells were randomly picked by FACS sorting per condition. Cells were allocated into experimental groups based on allele-specific chrX expression levels after excluding the top 10% expressed chrX genes to avoid bias from highly expressed genes. Due to differences in X-controlling elements (Xce) between C57BL6/J and CAST/EiJ strains, the C57BL6/J allele is preferentially inactivated during random X-inactivation. We did not control for this covariate as it had no noticeable effect in our analyses.
Blinding	Cells were blindly picked by FACS sorting per condition. Investigators were blinded to group allocation during data collection, but blinding was not possible during analysis due to data-driven group allocations.

Reporting for specific materials, systems and methods

We require information from authors about some types of materials, experimental systems and methods used in many studies. Here, indicate whether each material, system or method listed is relevant to your study. If you are not sure if a list item applies to your research, read the appropriate section before selecting a response.

Materials & experimental systems

n/a	Involved in the study	
☒	☐	Antibodies
☐	☒	Eukaryotic cell lines
☒	☐	Palaeontology and archaeology
☐	☒	Animals and other organisms
☒	☐	Human research participants
☒	☐	Clinical data
☒	☐	Dual use research of concern

Methods

n/a	Involved in the study	
☒	☐	ChIP-seq
☒	☐	Flow cytometry
☒	☐	MRI-based neuroimaging

Eukaryotic cell lines

Policy information about cell lines

Cell line source(s)	mESCs derived at the research institute.
Authentication	Cell identity confirmed by the RNA-sequencing (F1 cells containing and expression known parental SNP patterns)
Mycoplasma contamination	Cells tested negative to mycoplasma by PCR analysis.
Commonly misidentified lines (See ICLAC register)	None.

Animals and other organisms

Policy information about studies involving animals; ARRIVE guidelines recommended for reporting animal research

Laboratory animals	Mus musculus. Strains: C57BL/6J and CAST/EiJ pure lines and F1 intercross. Mice were housed in specific pathogen-free at Comparative Medicine Biomedicum (KM-B) according to Swedish national regulations for laboratory animal work food and water ad libitum, cage enrichment, and 12 hours light and dark cycles (ethical permit 17956-2018 and 18729-2019, Jordbruksverket). Embryonic stem cells were derived from E4 F1 blastocysts and liver tissue was isolated from 12-week-old male F1 mice. For the developmental analysis of XCU and XCU the embryonic day is specified in each analysis in the article.
Wild animals	None.
Field-collected samples	None.
Ethics oversight	All animal experimental procedures were performed in accordance with Karolinska Institutet's guidelines and approved by the Swedish Board of Agriculture (permits 17956-2018 and 18729-2019 Jordbruksverket).

Note that full information on the approval of the study protocol must also be provided in the manuscript.
